# The difference in the cellular uptake of tocopherol and tocotrienol is influenced by their affinities to albumin

**DOI:** 10.1038/s41598-023-34584-z

**Published:** 2023-05-06

**Authors:** Takashi Nakatomi, Mayuko Itaya-Takahashi, Yosuke Horikoshi, Naoki Shimizu, Isabella Supardi Parida, Mirinthorn Jutanom, Takahiro Eitsuka, Yoshikazu Tanaka, Jean-Marc Zingg, Tatsuya Matsura, Kiyotaka Nakagawa

**Affiliations:** 1 Laboratory of Food Function Analysis, Graduate School of Agricultural Science, Tohoku University, 468-1 Aramaki Aza Aoba, Aoba-ku, Sendai, 980-8572 Japan; 2 Division of Medical Biochemistry, Department of Pathophysiological and Therapeutic Sciences, Tottori University Faculty of Medicine, 86 Nishi-cho, Yonago, 683-8503 Japan; 3 Applied Biological Molecular Science, Graduate School of Life Sciences, Tohoku University, 2-1-1 Katahira, Aoba-ku, Sendai, 980-8577 Japan; 4 Department of Biochemistry and Molecular Biology, University of Miami, 1011 NW 15th St, Miami, FL 33136-1019 USA; 5 Department of Nutritional Sciences, Faculty of Human Ecology, Yasuda Women’s University, 6-13-1 Yasuhigashi, Asaminami-ku, Hiroshima, 731-0153 Japan

**Keywords:** Lipids, Cell biology, Lipids, Blood proteins, Carrier proteins, Chemical biology, Blood proteins, Carrier proteins, Biochemistry, Molecular modelling, Molecular modelling

## Abstract

Vitamin E is classified into tocopherol (Toc) and tocotrienol (T3) based on its side chains. T3 generally has higher cellular uptake than Toc, though the responsible mechanism remains unclear. To elucidate this mechanism, we hypothesized and investigated whether serum albumin is a factor that induces such a difference in the cellular uptake of Toc and T3. Adding bovine serum albumin (BSA) to serum-depleted media increased the cellular uptake of T3 and decreased that of Toc, with varying degrees among α-, β-, γ-, and δ-analogs. Such enhanced uptake of α-T3 was not observed when cells were incubated under low temperature (the uptake of α-Toc was also reduced), suggesting that Toc and T3 bind to albumin to form a complex that results in differential cellular uptake of vitamin E. Fluorescence quenching study confirmed that vitamin E certainly bound to BSA, and that T3 showed a higher affinity than Toc. Molecular docking further indicated that the differential binding energy of Toc or T3 to BSA is due to the Van der Waals interactions via their side chain. Overall, these results suggested that the affinity of Toc and T3 to albumin differs due to their side chains, causing the difference in their albumin-mediated cellular uptake. Our results give a better mechanistic insight into the physiological action of vitamin E.

## Introduction

**Figure 1:**
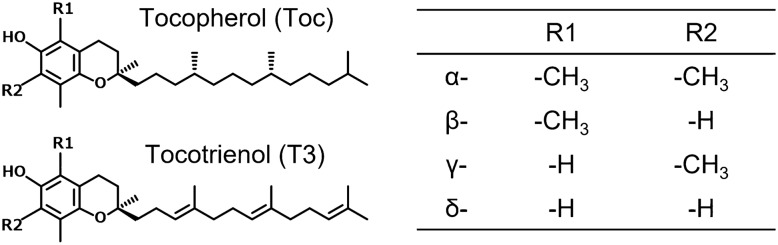
Chemical structure of vitamin E.

Vitamin E is a generic name that refers to two tocochromanols, tocopherol (Toc) and tocotrienol (T3) (Fig. 1). Toc and T3 differ in their side chain structures; whereas Toc has a saturated phytyl side chain attached to its chromanol ring, T3 possesses an unsaturated isoprenoid side chain. Additionally, Toc and T3 analogs are divided into their α-, β-, γ-, and δ-analogs in terms of the numbers and positions of methyl groups on their chromanol rings^1^. Toc is widely contained in lipid-rich plant products and common vegetable oils, and T3 is particularly abundant in some foods such as cereals, rice bran oil, and palm oil^2^. The estimated daily intake from foods of Toc and T3 is 8–10 mg/day and 1.9–2.1 mg/day per person, respectively^3^.

In recent years, T3 has attracted scientific interest, because it has shown higher physiological activities than Toc in various cell culture studies. For instance, T3 has been reported to exhibit higher antioxidative^4,5^, cholesterol-lowering^6,7^, and anti-cancer^8^ activities than Toc. Furthermore, we have previously revealed that T3 has more potent anti-cancer activities (i.e., anti-angiogenic^9–11^ and telomerase inhibitory^12,13^ effects) compared to Toc. In various cell culture experiments, T3 has shown a higher cellular uptake than Toc^14–24^, which is considered the reason for the superior physiological activity of T3. Indeed, in Jurkat cells, α-T3 has been reported to exhibit a 6.5-fold higher cellular uptake than α-Toc after incubation with 1 μM α-Toc or α-T3 for 24 hours^14^. Furthermore, in LNCaP and PC-3 cells, γ-T3 has demonstrated a higher cellular uptake than γ-Toc even after incubation with 50 μM γ-Toc or 10 μM γ-T3 for 6 hours^19^. However, the detailed mechanism by which T3 is able to demonstrate a higher cellular uptake than Toc remains unclarified.

In this study, we focused on the affinity of vitamin E analogs to albumin and explored why the cellular uptake of T3 is higher than that of Toc. Albumin is a major protein in fetal bovine serum (FBS), usually added to the cell culture medium. It is generally considered that the cellular uptake of various food and drug compounds is affected by their affinities to albumin, as shown in previous studies as well as our recent studies^25–29^. With regard to vitamin E, a previous in silico analysis predicted that the affinity of T3 to albumin is higher than that of Toc^30^. Hence, such differences in their affinities to albumin might have an influence on the cellular uptake of Toc and T3; however, this has not been investigated previously.

To investigate the above, we evaluated the incorporation of Toc and T3 into cells using culture medium that contained different concentrations of albumin. Consequently, albumin seemed to affect the difference in the cellular uptake between Toc and T3. Hence, we analyzed the affinity of Toc and T3 to albumin by fluorescence spectroscopy and a computational molecular docking approach^30–36^. As a result, we found that Toc and T3 demonstrate different affinities to albumin, and this difference might have induced the difference in their cellular uptake. We anticipate that these findings can further contribute to a better understanding of the physiological activities of vitamin E.

## Material and methods

### Materials and cells

All Toc analogs (α-, β-, γ-, and δ-Toc) and T3 analogs (α-, β-, γ-, and δ-T3) were kindly provided by Mitsubishi Chemical Corporation, Ltd. (Tokyo, Japan). These were all d-forms and were individually dissolved in dimethyl sulfoxide (DMSO) to prepare stock solutions at a concentration of 20.0 mM. Bovine serum albumin (BSA; Fatty Acid/IgG/Protease-Free) and phosphate-buffered saline (PBS) were purchased from FUJIFILM Wako Pure Chemical Corporation, Ltd. (Osaka, Japan). BSA was dissolved in PBS to prepare a 2.0 mM stock solution. BSA was also dissolved in 10.0 mM sodium phosphate buffer (pH 7.4) to prepare a 2.0 μM stock solution. All other chemicals and reagents used were of analytical grade or higher.

The human acute monocytic leukemia cell line (THP-1) was obtained from Riken Cell Bank (Tsukuba, Japan). THP-1 monocytes were cultured in RPMI (Roswell Park Memorial Institute; NY, USA) medium supplemented with 10% fetal bovine serum (FBS; Thermo Fisher Scientific, MA, USA), 100.0 U/mL penicillin, and 100.0 μg/mL streptomycin in an incubator at 37 °C containing 5% CO_2_ under humidified atmosphere.

### Treatment of THP-1 monocytes with vitamin E

THP-1 monocytes (2.5 × 10^6^ cells) were preincubated in 5.0 mL of 10% FBS/RPMI medium. The cells (2.5 × 10^6^ cells) were also preincubated in 5.0 mL of FBS-free 10% PBS/RPMI medium in the absence or presence of 0.2, 2.0, 20.0, 32.4, and 200.0 μM BSA (each medium was prepared by dissolving the 2.0 mM BSA stock solution in 10% PBS/RPMI medium). After 24 h of these preincubations, 5.0 μL of each vitamin E stock solution was added to 10% FBS/RPMI medium or 10% PBS/RPMI medium at a final concentration of 20.0 μM and further incubated for 2 h. The final concentration of DMSO was 0.1% (v/v), which did not affect cell viability. The incubated cells were collected by centrifugation and washed with PBS. The cells were resuspended in 0.6 mL water and homogenized on ice. A part of the lysate (0.5 mL) was used to analyze vitamin E (described below), and the remaining 0.1 mL was used to quantify protein by the BCA Protein Assay (Thermo Fisher Scientific).

### Treatment of THP-1 monocytes with α-Toc or α-T3 at low temperature (4 °C) where the uptake pathway is limited to passive diffusion

THP-1 monocytes preincubated in 10% FBS/RPMI medium were collected. The collected cells (2.5 × 10^6^) were suspended in 1.0 mL of 10% PBS/RPMI medium containing 0, 0.2, 2.0, 20.0, 32.4, and 200.0 μM BSA on ice. This suspension was immediately mixed with 4.0 mL of 10% PBS/RPMI medium containing BSA and vitamin E. The final composition was 5.0 mL of 10% PBS/RPMI medium containing 0, 0.2, 2.0, 20.0, 32.4, or 200.0 μM BSA, and 20.0 μM α-Toc or α-T3. Cells were incubated at 4 °C with slow rotation in a tube rotator. After 2 h incubation, the cell lysate was prepared as above, and vitamin E and protein levels were measured.

### Analysis of vitamin E

Vitamin E was extracted from the cell lysates according to the procedure described in our previous study^37^. Tocol (Tama Biochemical, Tokyo, Japan) was used as an internal standard. The extract was analyzed by liquid chromatography-tandem mass spectrometry (LC–MS/MS). The LC–MS/MS system consisted of an ExionLC system and a 4000QTRAP mass spectrometer (SCIEX, Tokyo, Japan). An Inersil SIL-100A-5 column (4.6 mm × 250 mm; GL Science, Tokyo, Japan) was eluted with a mobile phase consisting of hexane/1,4-dioxane/2-propanol (100:4:0.5, v/v/v). The flow rate was 1.0 mL/min, and the column temperature was 40 °C. Vitamin E was detected in the multiple reaction monitoring (MRM) mode (Table 1) under atmospheric pressure chemical ionization. The ion source parameters were as follows: curtain gas, 45.0 psi; collision gas, 8.0 psi; nebulizer current, − 3.0 μA; temperature, 350.0 °C; ion source gas 1, 60.0 psi; interface heater, ON.

**Table 1:** Multiple-reaction monitoring parameters for vitamin E analysis.

	α-Toc	β-Toc	γ-Toc	δ-Toc	α-T3	β-T3	γ-T3	δ-T3	Tocol
Precursor ion (*m/z*)	429.2	415.1	415.1	401.3	423.1	409.3	409.3	395.2	387.3
Product ion (*m/z*)	162.4	148.6	148.6	134.9	162.4	148.9	148.9	134.9	120.9
Declustering potential (V)	− 95	− 95	− 95	− 90	− 85	− 95	− 95	− 85	− 105
Entrance potential (V)	− 10	− 10	− 10	− 14	− 10	− 14	− 14	− 14	− 10
Collision energy (V)	− 36	− 34	− 34	− 36	− 34	− 34	− 34	− 36	− 60
Collision cell exit potential (V)	− 11	− 5	− 5	− 3	− 5	− 7	− 7	− 3	− 10

### Binding parameters between vitamin E and BSA

The binding parameters of each Toc or T3 analog to BSA were measured with slight modifications to a previously described procedure^32,34^. Each Toc or T3 analog was dissolved in DMSO at 10 mM, and added to a quartz cell containing 3.0 mL of 2.0 μM BSA stock solution to achieve a final concentration of 1.0–225.0 μM. The concentration of DMSO was adjusted to less than 2.25%, which does not to affect the native structure and binding properties of BSA^38^. Fluorescence spectra were recorded at room temperature using FP-6200 (JASCO, Tokyo, Japan) at an excitation wavelength of 280 nm and emission wavelengths from 300 to 500 nm. The intensity at 340 nm was used to calculate the Stern–Volmer constant (*K*_sv_) by the following equation^39^: *F*_0_/*F* = 1 + *K*_sv_[VE]. We also calculated the apparent binding constant (*K*_a_) and the number of binding sites (*n*) by the following equation^40^: log((*F*_0_ − *F*)/*F*) = *n*log*K*_a_ − *n*log(1/([VE] − [BSA](*F*_0_ − *F*)/*F*_0_)). Abbreviations in the above equations refer to follows: *F*_0_, initial fluorescence intensity; *F*, fluorescence intensity in the presence of each Toc or T3 analog; [VE], molar concentration of each Toc or T3 analog; [BSA], original BSA concentration. The same measurements were performed using a buffer solution without BSA, and the *F* values were calculated using the measured fluorescence intensity as a blank.

### Molecular docking simulation between vitamin E and BSA

With regard to the ligands, 3D structures of Toc and T3 analogs were obtained as SDL files from PubChem (PubChem CID: 14,985 (α-Toc), 6,857,447 (β-Toc), 92,729 (γ-Toc), 92,094 (δ-Toc), 5,282,347 (α-T3), 5,282,348 (β-T3), 5,282,349 (γ-T3), and 5,282,350 (δ-T3))^41^, and converted into a PDB file using the PyMOL software (Schrödinger, NY, USA). The 3D structures of the side chains of Toc (2,6,10-trimethyl-tridecane) and T3 (2,6,10-trimethyl-(E, E)-tridecatriene) were drawn using the MarvinSketch software (ChemAxon, Budapest, Hungary), and saved as a PDB file. To prepare a ligand file for docking calculations, the above PDB files were saved into a PDBQT file using the AutoDockTools (ADT) software version 1.5.6^42^ by detecting the torsion tree root of each ligand. As for the protein, the 3D structure of BSA (dimer), determined by x-ray crystallography, was downloaded from the RCSB Protein Data Bank (https://www.rcsb.org) (PDB ID: 4F5S^33^). PyMOL was used to remove co-crystallized compounds, water molecules, and one of the two BSA molecules in an asymmetric unit from the BSA structure. Polar hydrogens were added to the remaining BSA monomer. Using ADT, flexible amino acid residues of BSA were set as follows: Lys204, Trp213, Arg217, Leu218, Lys221, Leu233, Leu259, Ile263, Ile289, and Ala290. A PDBQT file for docking calculations was created using these flexible amino acid residues that comprise the drug site I of BSA^33,43^. Another PDBQT file for the rigid protein that represented the entire BSA structure was created. Using PDBQT files of the ligand, the flexible residues, and the rigid protein, molecular docking calculations were performed by the AutoDock Vina software^44^. Calculations were carried out by setting up the grid box with the size of x = 18 Å, y = 32 Å, and z = 18 Å, and with its center at x = − 3.162, y = 12.028, and z = 103.056, to include all above flexible residues, and by setting the exhaustiveness value to 8. Orientations with the lowest docking energy (the highest score) was selected as the most suitable docking model. The results of the molecular docking were visualized using PyMOL.

### Other cell culture assay

To further investigate cellular uptake mechanism, cell culture study was conducted using two ATP synthesis inhibitors as follows: (1) 1 mM 2,4-dinitrophenol or (2) a mixture of 10 mM sodium azide and 5 mM 2-deoxy-d-glucose^45^. THP-1 monocytes (2.5 × 10^6^) preincubated in 10% FBS/RPMI medium were collected, suspended in 1.0 mL of PBS(+)^45^ containing each inhibitor, and preincubated for 10 min at 37 °C under slow rotation in a tube rotator. After preincubation, 4.0 mL of PBS( +) containing BSA, vitamin E (δ-Toc or δ-T3) and each inhibitor was added (final concentrations of BSA and vitamin E were 10.0 μM), and cells were incubated for 1 h at 37 °C under slow rotation. Control samples were similarly prepared without the addition of inhibitors and incubated for 1 h. Vitamin E and protein levels were measured as described above.

### Statistical analysis

Experiments were conducted in triplicate. The results of all experiments are expressed as mean ± standard error (SE). The results of 2–2. and 2–3. were analyzed using one-way ANOVA, followed by post-hoc multiple comparisons by the Tukey–Kramer test. The results of 2–7. were analyzed using one-way ANOVA, followed by post-hoc multiple comparisons by the Dunnett’s test. Differences with *p* < 0.05 were considered statistically significant.

## Results and discussion

### Effect of serum on the cellular uptake of Toc and T3

**Figure 2:**
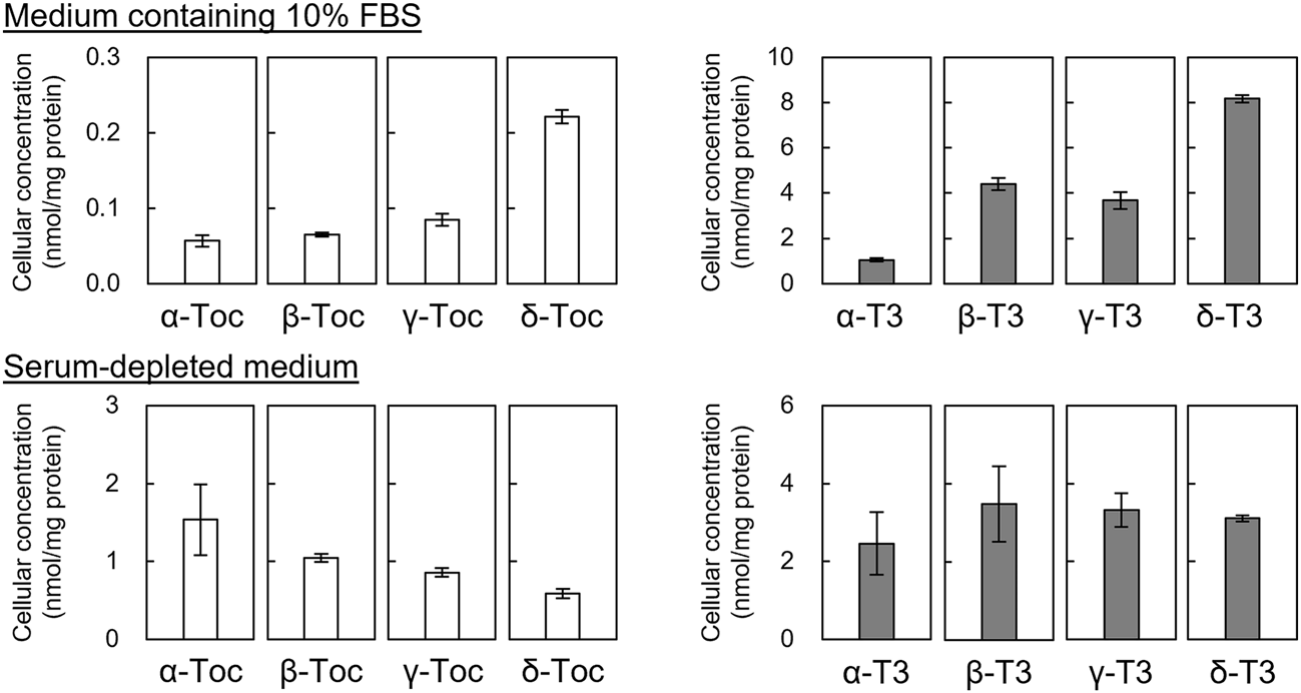
The effect of serum on the cellular uptake of Toc and T3 analogs in THP-1 monocytes. The cells were incubated with 20.0 μM of each analog in medium containing 10% FBS or serum-depleted medium for 2 h. The cellular concentrations of Toc and T3 were measured by LC–MS/MS. Values are expressed as mean ± SE of three independent experiments.

Previous cell culture experiments have observed that T3 is more readily incorporated into cells than Toc under various culture conditions^14–24^. The most likely reasoning for such difference in the cellular uptake of Toc and T3 is their difference in the passive diffusion across the membrane^14,15,46^; in vitro studies have reported that T3 has a higher intermembrane mobility than Toc^4,47^. On the other hand, some components of cell culture medium (e.g., serum) are known to affect the cellular uptake of various food and drug compounds^28,48,49^. Thus, we scrutinized the medium conditions of previous studies and noticed that various culture medium conditions, including that with serum and without serum^14–24^, were used. In consideration of this fact, we attempted to evaluate whether serum affects the cellular uptake of vitamin E. This was done by comparing the uptake of Toc and T3 using THP-1 monocytes cultured in medium with or without FBS. When the respective analogs of Toc or T3 (20.0 μM each) were incubated with THP-1 monocytes in medium containing 10% FBS for 2 h, T3 concentrations in the cells ranged from 1.05 to 8.17 nmol/mg protein, depending on the analog added (Fig. 2). These concentrations were certainly higher than Toc analog concentrations (0.06–0.22 nmol/mg protein) in the cells. Meanwhile, a different trend was observed when THP-1 monocytes were cultured in serum-depleted medium; T3 concentrations in the cells ranged from 2.46 to 3.48 nmol/mg protein and Toc concentrations ranged from 0.59 to 1.54 nmol/mg protein (Fig. 2). These results suggested that the cellular uptake among Toc and T3 analogs may differ depending on the presence or absence of serum in the cell culture medium. These data led us to hypothesize that serum components (e.g., albumin, a major serum protein), in addition to the already known passive diffusion^14,15,46^, is a factor that in-duces the difference in the cellular uptake of Toc and T3.

### Effect of albumin on the difference in the cellular uptake of Toc and T3

**Figure 3:**
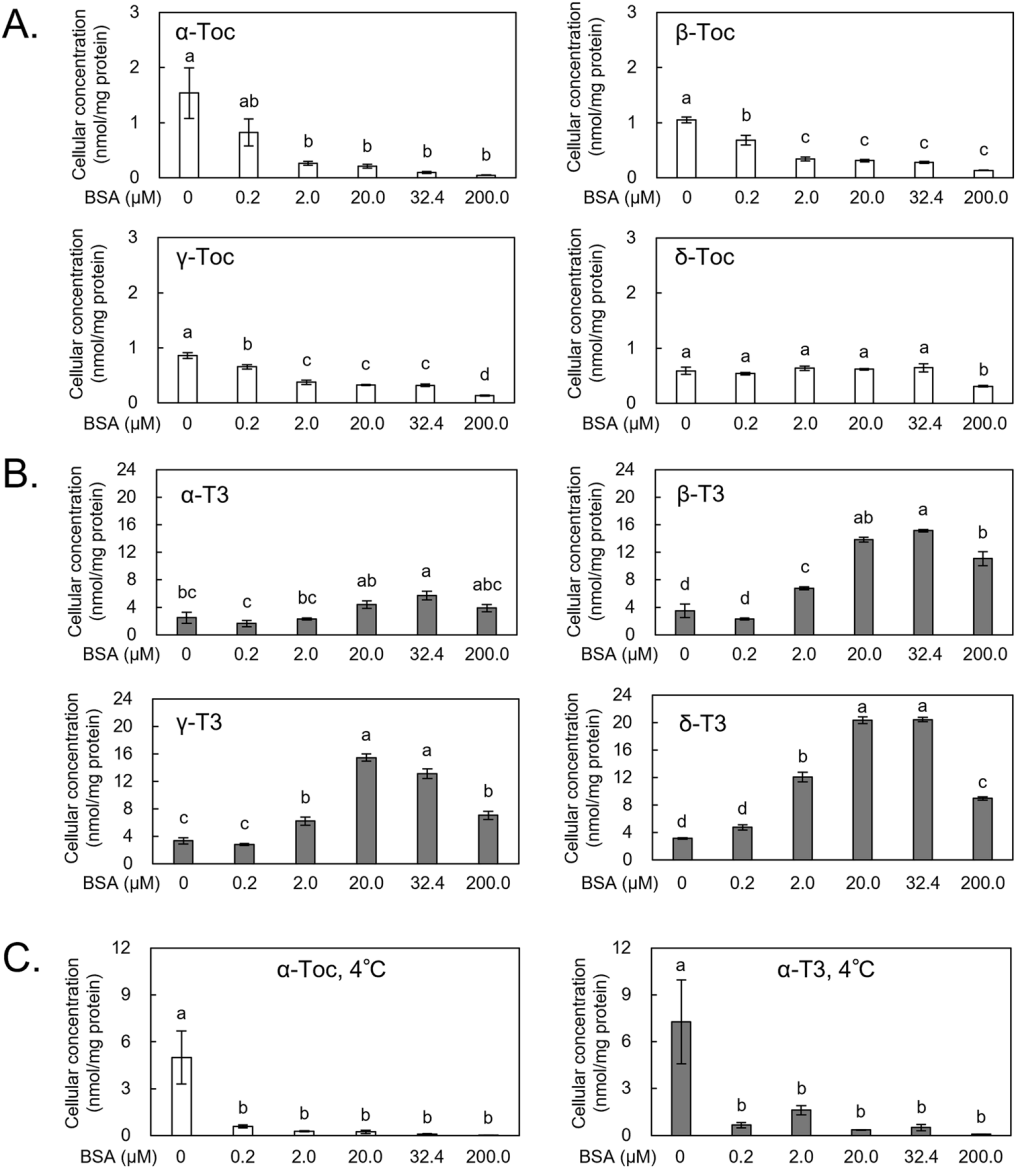
The effect of BSA on the cellular concentration of Toc and T3 analogs in THP-1 monocytes. The cells were incubated with 20.0 μM of a Toc (**A**) or T3 (**B**) analog in serum-depleted medium containing 0, 0.2, 2.0, 20.0, 32.4, or 200.0 µM of BSA for 2 h. Note that the value at 0 μM BSA is the same data as the serum-depleted medium in Fig. 2. The cells were incubated at low temperature (4 °C) with 20.0 μM of α-Toc or α-T3 in the serum-depleted medium containing the indicated concentrations of BSA for 2 h (**C**). The cellular concentrations of Toc and T3 were measured by LC–MS/MS. Values are expressed as mean ± SE of three independent experiments. Different letters indicate significant differences (*p* < 0.05).

As mentioned above, we focused on albumin as a serum component that affects the cellular uptake of Toc/T3 and prepared serum-depleted medium containing various concentrations of BSA (0, 0.2, 2.0, 20.0, 32.4, and 200.0 μM, as well as 32.4 μM which is equivalent to the albumin concentration of a medium containing 10% FBS). In each medium, THP-1 monocytes were cultured with a Toc or T3 analog (20.0 μM) for 2 h to examine the effect of albumin on the cellular uptake of vitamin E. In the case of T3, the addition of BSA to serum-depleted medium significantly increased cellular T3 concentrations (Fig. 3B). For most T3 analogs, maximum T3 concentrations (5.67–20.43 nmol/mg protein) were reached by the addition of 32.4 μM BSA. Comparing each analog, δ-T3 was found to increase the most, followed in order by β-T3, γ-T3, and α-T3. Meanwhile, a contrasting pattern was observed for Toc (Fig. 3A). The addition of BSA to the serum-depleted medium reduced Toc concentration in the cells, which was particularly pronounced for α-Toc, followed by β-Toc, γ-Toc, and δ-Toc. Such differences in cellular uptake of Toc and T3 in response to BSA addition were more evident than that observed in the study where the addition of FBS was evaluated (Figs. 2 and 3A,B), thereby strongly suggesting that the albumin content in serum plays an important role in this difference. Perhaps, albumin also influences the difference in the cellular uptake of their α-, β-, γ-, and δ-analogs.

### Possible formation of vitamin E-albumin complex and its cellular uptake pathway

Regarding the binding of food and drug compounds to albumin, there are two common pathways by which compounds are taken up into cells via albumin complexes. One pathway is the passive diffusion of compounds released from the albumin complex, in which compounds with lower affinity to albumin are believed to be more likely taken up by cells^28,48,49^. In the other pathway, the binding of compounds to albumin promotes their uptake into cells, as is the case for fatty acids. In this particular mechanism, compounds enter cells either through the endocytosis of the albumin-compound complex^49–52^ or via facilitated diffusion following the dissociation of compounds from the albumin complexes upon the binding of complexes to cell surface receptors^26,27,53,54^. Given the results of this study (i.e., the addition of BSA to serum-depleted medium enhanced the cellular uptake of T3 and decreased the uptake of Toc; Fig. 3A,B), the difference in their cellular uptake is likely due to the latter pathway (via cell surface receptors).

To confirm the above premise, we investigated the cellular uptake of α-Toc and α-T3 at a low temperature (4 °C). Under this condition, we did not observe the BSA-induced increase in α-T3 uptake that was previously notable under incubation at 37 °C (Fig. 3B,C). Meanwhile, the cellular uptake of α-Toc was reduced upon the addition of BSA regardless of the incubation temperature (Fig. 3A,C). Considering that compounds are generally taken up by cells by passive diffusion under such low-temperature conditions^55,56^ and the fact that the cellular uptake of α-Toc and α-T3 was reduced in response to the addition of BSA under low temperature, we speculate that Toc and T3 can bind to albumin, forming a complex that prevents the passive diffusion of Toc and T3.

Incubation under such low temperature is also useful to assess the binding of albumin complexes to the cell surface (e.g., via albumin-specific receptor)^43,57^. In this study, the cellular concentration of α-T3 was slightly higher than that of α-Toc when each was incubated in BSA-containing medium under low temperature (Fig. 3C), which indicated that the albumin-T3 complex is more likely to bind to the cell surface than the albumin-Toc complex. To further investigate these possibilities mentioned above, we believed it was crucial to examine the binding affinity of vitamin E to albumin.

### Evaluation of the affinity of Toc and T3 to BSA by the fluorescence quenching technique

**Figure 4:**
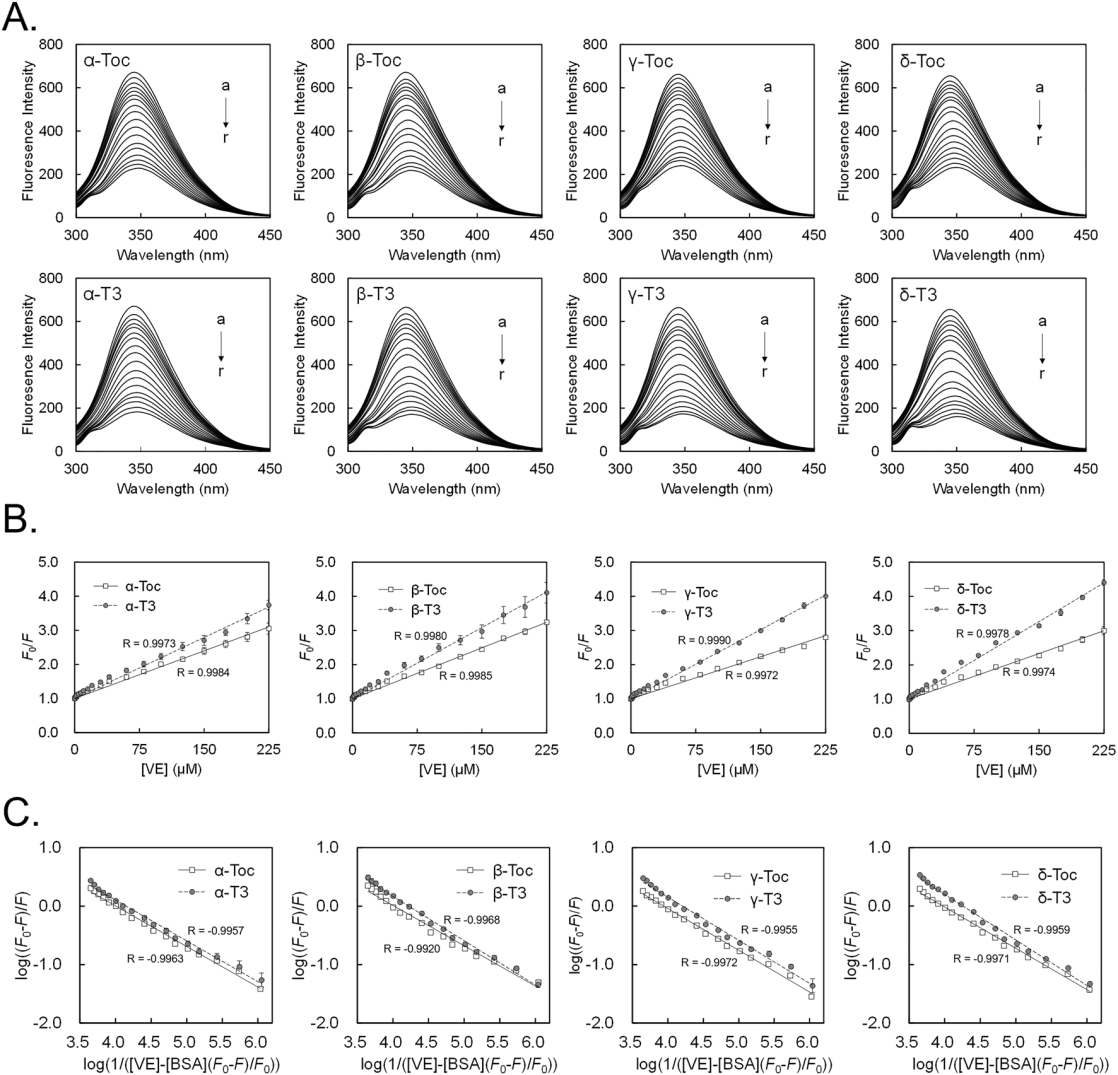
Fluorescence quenching study of BSA with vitamin E. Fluorescence emission spectra of a 2.0 μM BSA in the presence of vitamin E were measured under the excitation wavelength of 280 nm (**A**). Each spectrum was recorded with the adjusted concentrations of each Toc or T3 analog at 0, 1.0, 2.0, 4.0, 7.0, 10.0, 15.0, 20.0, 30.0, 40.0, 60.0, 80.0, 100.0, 125.0, 150.0, 175.0, 200.0, and 225.0 μM (a–r). The plots of *F*_0_/*F* as a function of [VE] (Stern–Volmer plot) (**B**) and the plots of log((*F*_0_ − *F*)/*F*) as a function of log(1/([VE] − [BSA](*F*_0_ − *F*)/*F*_0_)) (modified Stern–Volmer plot) (**C**) are shown. Data are expressed as mean ± SE of three independent experiments.

To examine the specific binding properties of each Toc or T3 analog to BSA, we determined their binding parameters using the fluorescence quenching technique. By comparing the fluorescence spectra (Fig. 4A), we found that T3 quenches the intrinsic fluorescence of BSA to a greater level than Toc at each concentration. Consequently, the Stern–Volmer quenching constants (*K*_sv_) and apparent binding constants (*K*_a_) of BSA-T3 were greater than those for BSA-Toc, and the constants for each analog were in the following order: δ-T3 > β-T3 > γ-T3 > α-T3 > β-Toc > α-Toc > δ-Toc > γ-Toc (Table 2, Fig. 4B,C). These results suggest that T3 certainly has a higher affinity for albumin than Toc; furthermore, their affinities also differ depending on their respective analogs (i.e., α-, β-, γ-, and δ-analogs).

**Table 2:** Stern–Volmer constants (*K*_sv_), apparent binding constants (*K*_a_) and the number of binding site (*n*) of vitamin E to BSA.

Vitamin E	*K*_sv_ [× 10^4^ M^−1^]	*K*_a_ [× 10^4^ M^−1^]	*n*
α-Toc	0.93 ± 0.06	1.01 ± 0.08	0.69 ± 0.02
β-Toc	1.00 ± 0.02	1.05 ± 0.04	0.69 ± 0.01
γ-Toc	0.82 ± 0.01	0.87 ± 0.02	0.71 ± 0.02
δ-Toc	0.89 ± 0.04	0.95 ± 0.04	0.71 ± 0.01
α-T3	1.19 ± 0.07	1.44 ± 0.16	0.70 ± 0.04
β-T3	1.39 ± 0.13	1.68 ± 0.19	0.76 ± 0.00
γ-T3	1.35 ± 0.01	1.61 ± 0.04	0.74 ± 0.02
δ-T3	1.51 ± 0.02	1.81 ± 0.02	0.78 ± 0.01

Data are expressed as mean ± S.E. (n = 3).

For reference, the *K*_sv_ and *K*_a_ measured in this study were not in perfect agreement with previous reports (among the Toc/T3 analogs, only the binding constants for α-Toc and BSA or human serum albumin have been reported^31,32,34^). This may be due to different solvents used to dissolve vitamin E (DMSO in this study and ethanol in previous studies) or to different sources of BSA. The number of binding sites (*n*) of each Toc or T3 analog to BSA was calculated to be approximately 1 (Table 2, Fig. 4C), estimating the presence of one binding site for each Toc or T3 analog and BSA. To our knowledge, this is the first study to show the number of Toc (other than α-Toc^32,34^) or T3 that bind to albumin in this way.

As described above, we were able to characterize the specific binding properties of vitamin E and albumin using the fluorescence quenching technique, and we decided to further investigate the binding properties from a structural perspective using molecular docking simulations.

### Molecular docking simulation of Toc and T3 to BSA

**Figure 5:**
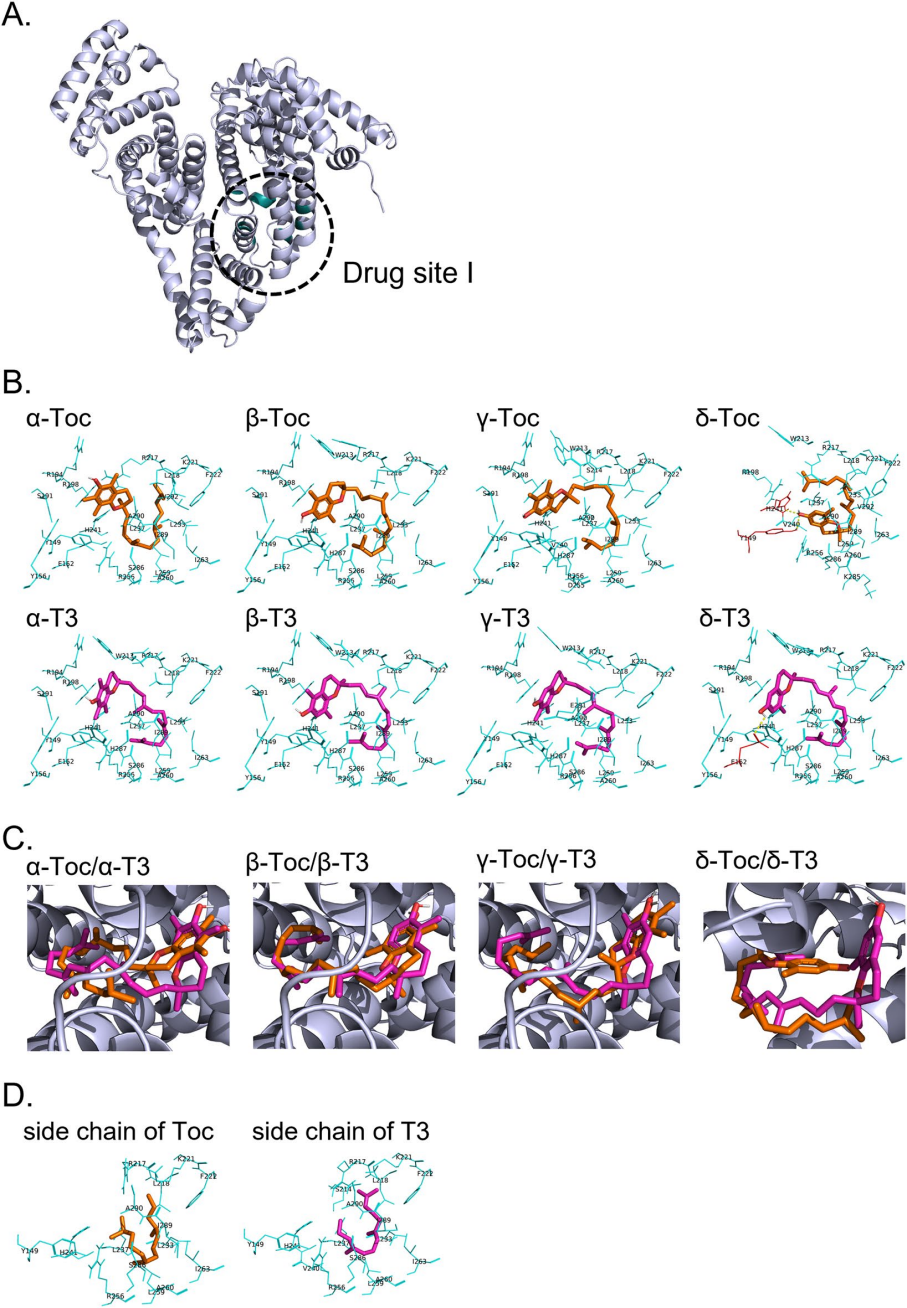
Molecular docking simulations of vitamin E (or its side chain) with BSA. The entire structure of BSA is represented as a cartoon and the drug site I is colored in dark cyan (PBD ID: 4F5S^33^) (**A**). Stereoviews of the docking models between each Toc or T3 analog and BSA (**B**), overlaid docking model with BSA for α-, β-, γ-, and δ-analogs (**C**), and stereoview of the docking models between each side chain of Toc or T3 and BSA (**D**) are shown. Toc and T3 (or their side chain) are represented in orange and magenta colors, respectively. Amino acid residues involved in Van der Waals interactions and hydrogen bonding are shown in cyan and red, respectively. Yellow demonstrates hydrogen bonds between an amino acid residue and the ligand.

The fluorescence from albumin used in the above experiments is considered to originate from Trp213 among the constituent amino acids of BSA. Since this Trp213 is a constituent of the drug site I of BSA, we tried to simulate the molecular docking of each Toc or T3 analog into this site (Fig. 5A)^33,58,59^. The docking models (Fig. 5B) suggested that all Toc and T3 analogs form Van der Waals interactions with 18–24 amino acid residues of BSA. An exception was the δ-analogs (δ-Toc and δ-T3) which formed hydrogen bonds with one or two amino acid residues. The binding energies of each Toc or T3 analog to BSA were in the following order: α-Toc > β-Toc > δ-Toc > γ-Toc > δ-T3 > α-T3 > γ-T3 > β-T3 (Table 3). This order was roughly consistent with that of the fluorescence quenching experiments described above (Table 2). Thus, in addition to fluorescence quenching experiments, the results of molecular docking simulations also suggested that T3 does indeed have a higher affinity for albumin than Toc.

**Table 3:** Calculated binding energies of vitamin E or its side chain to BSA.

Ligand	Binding energy to BSA [kcal/mol]
α-Toc	− 9.1
β-Toc	− 9.3
γ-Toc	− 9.8
δ-Toc	− 9.6
α-T3	− 10.3
β-T3	− 10.5
γ-T3	− 10.4
δ-T3	− 10.2
Side chain of Toc	− 7.2
Side chain of T3	− 8.0

In comparison of the Toc and T3 analogs with the same chromanol ring (e.g., α-Toc and α-T3), overlaid docking models with BSA showed that the side chains of Toc and T3 are in approximately the same position (Fig. 5C). We also simulated the molecular docking between each side chain (not vitamin E itself) and albumin. From the obtained docking models (Fig. 5D), both side chains were suggested to form Van der Waals interactions with 15 amino acid residues (Tyr149, Arg217, Leu218, Lys221, Phe222, Leu233, Leu237, His241, Arg256, Leu259, Ala260, Ile263, Ser286, Ile289, and Ala290). In general, Tyr149, Arg217, Lys221, Leu237, His241, Arg256, and Ala290 at the drug site I are considered to be involved in the binding of various compounds to BSA^59,60^. Therefore, the interaction of these amino acids with the vitamin E side chain may play an important role in the binding of Toc or T3 to BSA. On top of that, as expected, calculations from these docking simulations indicated that the T3 side chain has a more stable binding energy to BSA than the Toc side chain (Table 3). Taken together, the above results suggest that the binding of Toc or T3 analogs to albumin involves Van der Waals interactions, particularly due to their respective side chain structures. Furthermore, since the albumin-T3 complex is formed with a more stable binding energy than the albumin-Toc complex, the resulting difference in the affinity of Toc and T3 to albumin may induce differences in their cellular uptake.

We are currently performing additional docking studies using different software. For example, MOE software (Molecular Operating Environment; Chemical Computing Group, Montreal, Canada) was used to calculate the docking of α-Toc or α-T3 to BSA by setting the docking site to the same amino acid residues as the flexible residues in the AutoDock Vina calculations above (i.e., drug site I of BSA). As a result, comparison of the calculated binding energies suggested that α-T3 binds more stably to BSA than α-Toc (data not shown). In the next study, further docking studies will increase the reliability of our theory about the binding between vitamin E and albumin.

## Conclusion and perspectives

In this study, we have shown that albumin (a major serum protein) mediates the difference in the cellular uptake of Toc and T3. The observable higher uptake of T3 than Toc in serum-containing medium was markedly enhanced by the addition of albumin to serum-depleted medium, with an increase in T3 and a decrease in Toc. Under low temperature incubation, both cellular uptake of α-Toc and α-T3 was inhibited, suggesting that the cellular uptake of vitamin E occurs through the formation of a complex with albumin, followed by endocytosis or facilitated diffusion. From the fluorescence quenching study, the binding of vitamin E to albumin was confirmed, and T3 showed a higher affinity to albumin than Toc. Molecular docking simulation indicated that this difference is due to differential binding energy derived from the Van der Waals interactions via their side chains. These results suggested that forming albumin-T3 complex with a higher affinity than albumin-Toc complex in cell culture medium is key to the difference in the cellular uptake of Toc and T3, probably through endocytosis or facilitated diffusion.

In the above, we discussed the difference in the affinity of each vitamin E analog to albumin and its effect on the cellular uptake of Toc and T3. To further understand this mechanism (i.e., how the albumin-vitamin E complex affects the cellular uptake of vitamin E into the cell), an additional cell culture experiment was performed using ATP synthesis inhibitors to determine whether the uptake is mediated by endocytosis or by facilitated diffusion. As a result, ATP synthesis inhibitors did not affect the cellular uptake of vitamin E (Fig. S1), suggesting that the involvement of ATP-dependent uptake pathways (e.g., endocytosis) are minimally involved in the cellular uptake of albumin-bound vitamin E. Thus, we believe that ATP-independent facilitated diffusion most likely contributes to the transport of vitamin E via formation of the albumin-binding complex. Further experiments (e.g., identification of vitamin E carrier proteins involved in the facilitated diffusion) will verify this facilitated diffusion pathway.

Although there have been many reports that observed differences in the cellular uptake of Toc and T3, to our knowledge, this is the first report demonstrating that serum albumin plays a crucial role in their cellular uptake. The results of this study may have important implications for further elucidating the physiological mechanisms of vitamin E and its transfer into cells in tissues.

## Supplementary Information

Supplementary Figure S1.

## Data Availability

The datasets used and/or analyzed during the current study are available from the corresponding author on reasonable request.
