# Supplementary material for: The difference in the cellular uptake of tocopherol and tocotrienol is influenced by their affinities to albumin
Source: Sci Rep. 2023 May 6;13:7392. doi: 10.1038/s41598-023-34584-z (PMC10164177; doi:10.1038/s41598-023-34584-z)
Supplement: Supplementary file 1 — Supplementary Figure S1. [file 41598_2023_34584_MOESM1_ESM.pdf]

Supplementary Material

The Difference in the Cellular Uptake of Tocopherol and Tocotrienol is Influenced by their Affinities to Albumin

Takashi NAKATOMI[1], Mayuko ITAYA-TAKAHASHI[1], Yosuke HORIKOSHI[2], Naoki SHIMIZU[1], Isabella Supardi PARIDA[1], Mirinthorn JUTANOM[1], Takahiro EITSUKA[1], Yoshikazu TANAKA[3], Jean-Marc ZINGG[4], Tatsuya MATSURA[2,5], Kiyotaka NAKAGAWA[1,*]

[1]*Laboratory of Food Function Analysis, Graduate School of Agricultural Science, Tohoku University, 468-1 Aramaki Aza Aoba, Aoba-ku, Sendai, 980-8572, Japan*
[2]*Division of Medical Biochemistry, Department of Pathophysiological and Therapeutic Sciences, Tottori University Faculty of Medicine, 86 Nishi-cho, Yonago, 683-8503, Japan*
[3]*Applied Biological Molecular Science, Graduate School of Life Sciences, Tohoku University, 2-1-1 Katahira, Aoba-ku, Sendai, 980-8577, Japan*
[4]*Department of Biochemistry and Molecular Biology, University of Miami, 1011 NW 15th St, Miami, Florida, 33136-1019, United States*
[5]*Department of Nutritional Sciences, Faculty of Human Ecology, Yasuda Women's University, 6-13-1 Yasuhigashi, Asaminami-ku, Hiroshima, 731-0153, Japan*

[*]**Corresponding author:** Food Function Analysis Laboratory, Graduate School of Agricultural Science, Tohoku University, 468-1 Aramaki Aza Aoba, Aoba-ku, Sendai, 980-8572, Japan. Fax: +81-22-757-4417.
E-mail address: kiyotaka.nakagawa.c1@tohoku.ac.jp

Figure S1

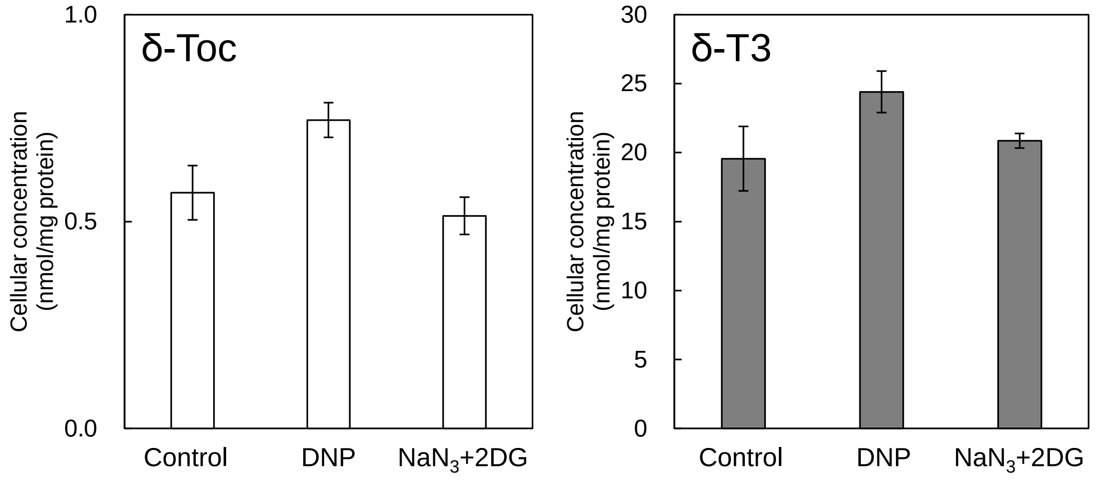

Figure S1 The effect of two ATP synthesis inhibitors (1 mM 2,4-dinitrophenol (DNP) or a mixture of 10 mM sodium azide and 5 mM 2-deoxy-D-glucose (NaN_3+2DG)) on the cellular uptake of δ-Toc and δ-T3 in THP-1 monocytes. Cells were incubated in PBS(+) containing 10.0 μM vitamin E (δ-Toc or δ-T3), 10.0 μM BSA, and each inhibitor for 1 hour. Control samples were similarly incubated without the addition of inhibitors. Cellular concentrations of δ-Toc and δ-T3 were measured by LC-MS/MS as described in the main article. Values are expressed as mean ± SE of three independent experiments. The statical significance of the results was analyzed using Dunnett's test, where $p < 0.05$ was considered statistically significant. No significance was observed between Control and samples treated with ATP synthesis inhibitor.
